# The management of Salter-Harris type II fracture with associated posterior sternoclavicular joint displacement using a locking compression plate: A 14-year-old adolescent's case report

**DOI:** 10.1097/MD.0000000000018433

**Published:** 2019-12-20

**Authors:** Matteo Vitali, Andreas Drossinos, Pierluigi Pironti, Elisa Pesce, Vincenzo Salini

**Affiliations:** a Department of Orthopedics and Traumatology, San Raffaele Scientific Institute; b Faculty of Medicine and Surgery, Vita-Salute San Raffaele University, Milan, Italy.

**Keywords:** displacement, fracture, internal stabilization, sternoclavicular joint

## Abstract

**Rationale:**

Posterior sternoclavicular joint dislocations (PSCJDs) are particularly rare injuries, accounting for 3% to 5% of sternoclavicular joint dislocations. With very few cases reported in the literature, these injuries are often misdiagnosed and imaging is not always clear, thus making physicians often unaware of them. The present case report aims to investigate a rare case involving a clavicular Salter-Harris II fracture with associated posterior displacement of the diaphysis, a term coined a “pseudodislocation.”

**Patient concerns:**

We present a case of a 14-year-old adolescent who sustained a traumatic injury to the shoulder while falling during a soccer match. His main concern was about recovery time and the return to daily life activities.

**Diagnoses:**

Multiple imaging studies imaging (X-rays, computed tomography, magnetic resonance imaging) revealed a Salter-Harris II fracture of the right clavicle with posterior displacement of the diaphysis.

**Interventions:**

The patient underwent primary surgery to reduce the fracture, using an articular locking compression plate, and secondary surgery to remove the hardware.

**Outcomes:**

Following the removal of the hardware at 60 days after the initial surgery and a number of cycles of physiotherapy the patient reported a pain-free range of motion with slight limitation at extremes. Full return to recreational and everyday life activities were achieved at 3 months from the initial surgery.

**Lessons:**

The PSCJDs are challenging injuries, as they are surrounded by delicate structures inside the mediastinum. Attention must be taken while diagnosing and treating these injuries as the risk of complications and iatrogenic injuries is high. To the author's knowledge, this case is one of the first of its kind described in the literature where we have a Salter-Harrys type II fracture associated with a posterior pseudodislocation of the lateral clavicle. Given the positive results of the case, we recommend the above-mentioned treatment protocol in PSCJD with associated Salter-Harris II fractures in adolescent patients.

## 1 Introduction

Posterior sternoclavicular joint dislocations (PSCJDs) are particularly rare injuries, comprising only 3% to 5% of sternoclavicular joint dislocations^[1]^ with very few cases reported in the literature. This type of injury is even rarer in adolescent patients, commonly occurring as a result of either a lateral force applied to the shoulder girdle or posteriorly directed force applied to the sternoclavicular joint (SCJ) directly^[2]^; or a combination of the 2 forces. In children, the physis is a structurally weak point due to skeletal immaturity, making a displaced metaphysis more common than a true sternoclavicular joint dislocation.^[3]^ Although there is still no consensus on the technical approach, reduction of the dislocation remains the guiding principle in the management of such injuries, with open reduction followed by internal fixation (ORIF) being the preferred treatment. To the author's knowledge, this is the 1st case concerning the management of an acute Salter-Harris type II fracture with associated posterior sternoclavicular joint displacement, also known as pseudodislocation, treated with a locking compression plate (LCP) across the SCJ in an adolescent patient.

## 2 Materials and methods

**Figure 1:**
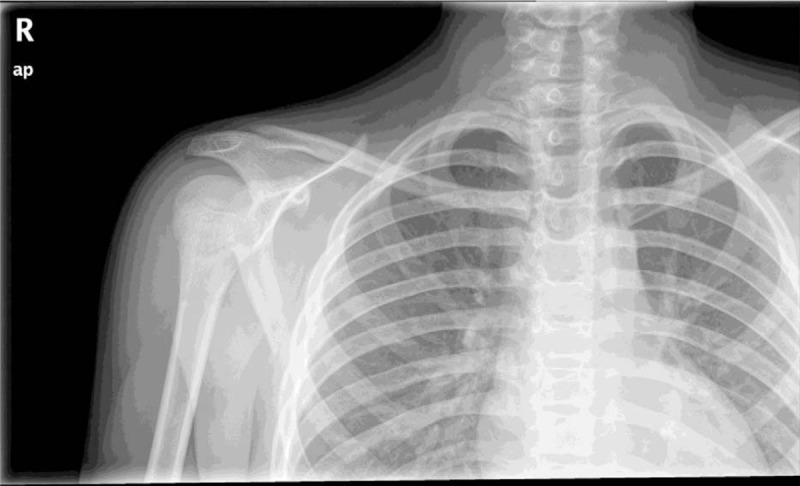
Anteroposterior thoracic X-rays.

**Figure 2:**
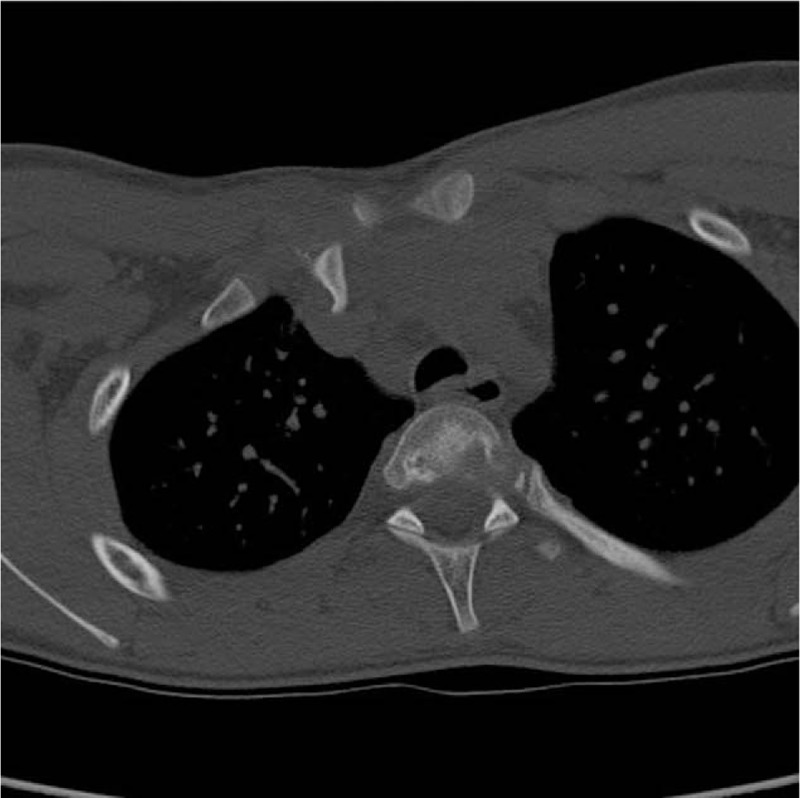
Computed tomography scan showing a right posterior sternoclavicular joint pseudodislocation.

**Figure 3:**
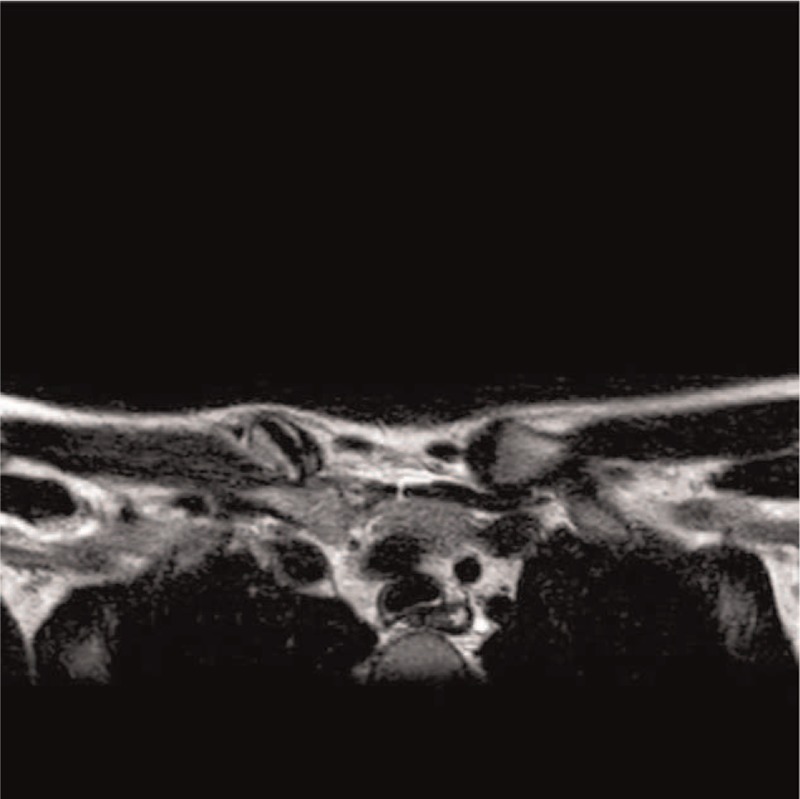
Magnetic resonance imaging showing a clavicular Salter-Harris type 2 fracture.

We present the case of a 14-year-old adolescent male patient who, during a soccer match, sustained a traumatic injury following a fall on the right shoulder. Physical examination showed a physical deformity over the right SCJ, with pain and functional limitation. No neurovascular deficit was present. There was no significant medical history, as well as familiarity to any medical condition. Anteroposterior thoracic X-rays confirmed the suspicion of asymmetry across the SCJ but did not allow for clear evaluation of the nature of the dislocation (Fig. 1). This warranted a computerized tomography (CT) scan that confirmed a right posterior SCJ pseudodislocation (Table 1, Fig. 2). While in the orthopedic ward, the patient underwent magnetic resonance imaging (MRI) which showed an additional epiphyseal fracture with evidence of a displaced small bone fragment containing a portion of the physis still covered by the growth plate cartilage (Salter-Harris type 2) (Table 1, Fig. 3). In addition, the medial clavicular head showed signs of mediastinal impingement, with adhesions observed between the clavicle and right innominate vein near the origin of the inferior thyroid vein. On the 3rd day upon admission, the patient was taken to the operating room to undergo surgical treatment of the SCJ through ORIF with LCP plate and screws. The patient was placed on beach chair position (BCP), and subsequently underwent a general anesthesia. A horizontal incision was made along the medial 3rd of the right clavicle, 2 cm from the center of the manubrium extending laterally for 4 cm. Intraoperatively, there was visible confirmation of the Salter-Harris II fracture associated with a posterior STJ pseudodislocation. The medial clavicle was reduced and stabilized by placing a titanium intra-articular Synthes LCP. To fix the LCP, five 2.7 mm Synthes cortex screws were positioned through the plate, 2 along the manubrium and 3 along the medial clavicle. The remnants of the posterior sternoclavicular ligament where reconstructed to further grant stability to the reduction. The patient was placed in a simple sling for 21 days after which X-rays were taken to assess the status of SCJ alignment (Table 1).

**Table 1:**
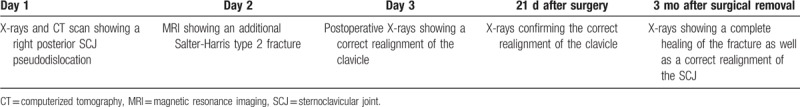
Radiologic findings.

**Figure 4:**
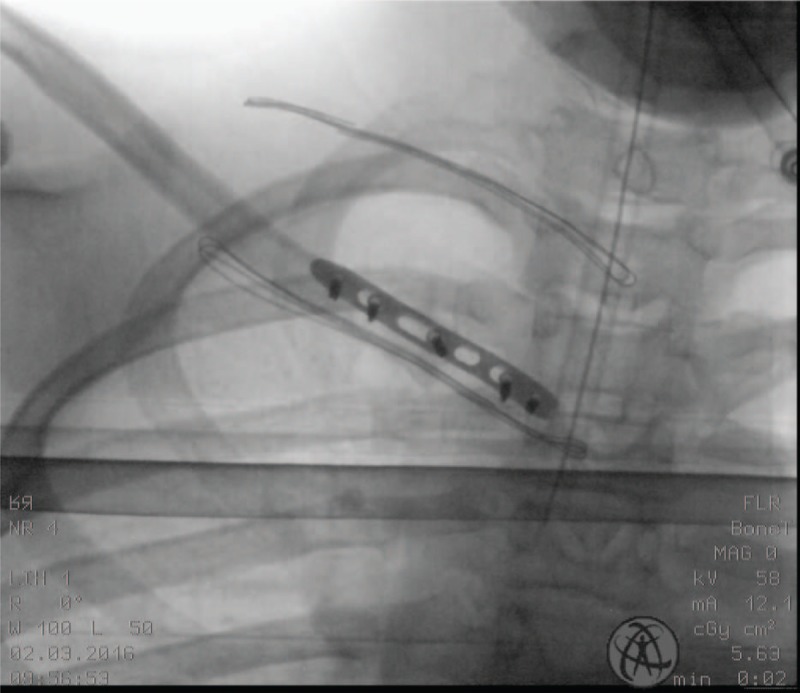
Postoperative imaging with plain radiographs showing correct realignment of the clavicle.

In the days following the 1st surgery with the LCP to reduce the pseudodislocation, the patient had very little to no pain. No adverse events, such as fever, infection, or hematoma, were experienced by the patient during the in-hospital stay. Postoperative imaging with plain radiographs showed correct realignment of the clavicle with respect to the manubrium and the contralateral clavicle in addition to maintenance of the reduction of the pseudodislocation (Fig. 4). The patient was discharged with the arm placed in a simple sling, which was removed after 21 days during a follow-up check in the outpatient clinic. While at home, the patient reported minimal pain at this stage of recovery with limited range of motion of the shoulder. At 60 days from the open reduction with SCJ stabilization, the patient underwent a 2nd surgical intervention to remove the inter-articular LCP. At 3 months from the surgical removal of the intra-articular plate, the patient was able to return to normal daily and athletic physical activities, with normal articular range of motion and good stability of the joint. Postoperative imaging with plain radiographs showed correct realignment of the sternoclavicular joint (Table 1). There were no adverse effects during the treatment. All diagnostic and interventional phases are summarized in Table 2.

**Table 2:**
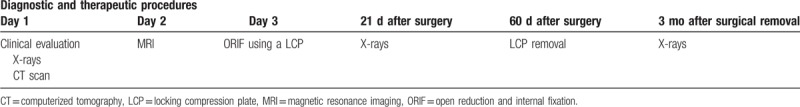
Timeline of diagnostic procedures and interventions.

This case description follows the principles of the declaration of Helsinki. The patient and his parents provided informed consent for publication of the case.

## 3 Discussion

Posterior sternoclavicular dislocations are particularly rare injuries^[1]^ with a low incidence that may be explained by misdiagnosis, lack of clear imaging associated with anteroposterior X-rays and general lack of awareness regarding the condition. Anterior dislocations are much more frequent given the inherent stability provided by the posterior capsule of the SCJ.^[4]^ This type of injury is growing in frequency in the adolescent population due to increased participation in contact sports combined with the skeletal immaturity of the SCJ in these young patients. In fact, the clavicle undergoes ossification and fusion with its physis at 18 to 20 and 22 to 25 years, respectively.^[5]^ For that reason, sternoclavicular joint dislocations are often not actual dislocations, but instead pseudo-dislocations, term coined by Robinson et al,^[6]^ where partial displacement occurs across the fractured physis and the SCJ remains unaltered.

In these instances, the SCJ remains intact with minimal or no lesion present on the stabilizing ligamentous structures. Instead, there is a disruption of the physeal plate with a slight displacement of the diaphysis with respect to the epiphysis. PSCJD usually occur as a result of indirect trauma^[7]^ wherein an anterolateral force is applied to the shoulder girdle, causing the lateral clavicle to move forward while the medial clavicle levers posteriorly. PSCJD resulting from direct traumas only account for 10% to 25% of SCJ injuries due to a posteriorly directed force applied to the SCJ, such as in an automobile accident.^[8]^ Patients typically present with pain, tenderness, and swelling of the SCJ as well as reduced range of motion of the ipsilateral arm.^[9]^ Treatment of such injuries may be managed more conservatively as the SCJ remains intact. Aydin and Metineren^[10]^ describe a case where the SCJ was stabilized with tension band technique using nonabsorbable sutures across the joint capsule. In our case, the patient's ligamentous capsule was compromised in addition to the Salter-Harris fracture across the physis of the medial clavicle as evidenced by imaging. Typical anteroposterior X-rays are of limited use as they produce obscure images due to the multitude of underlying soft tissues surrounding the SCJ.^[11]^ CT is the preferred imaging test performed, providing an axial representation of the SCJ with clear visualization of the surrounding tissues in case of mediastinal compression. Given the rarity of PSCJD, there is currently no consensus on the management of such injuries.^[12]^ Closed reductions are advised for patients presenting within 48 hours of injury,^[13]^ with success rates of 55.8%. Closed reductions performed after the initial 48 hours have reduced success rates of 30.8%.^[10]^ It must be noted that following a PSCJD, the integrity of the posterior capsular ligaments tends to be compromised. Since this structure is the strongest ligamentous stabilizer of the SCJ,^[14]^ it must be repaired after a reduction of the SCJ to avoid a joint instability. This helps to explain the elevated failure rates of closed reductions since they do not address this destabilizing lesion whatsoever. As such, Waters et al^[15]^ found open reduction with joint stabilization to be the best treatment option for skeletally immature patients with PSCJD and medial clavicular fractures. Several surgical techniques are described in the literature. Kirschner wires and Balser plates are no longer considered valid options for SCJ stabilization given their associated risks of intrathoracic migration^[16–18]^ and SCJ arthropathy, respectively. Specifically, Balser plate hooks inserted in the manubrium of the sternum reduce joint mobility and lead to damage of the articular processes.^[19]^ It has been reported that the optimal approach to dealing with PSCJDs is to 1st attempt a closed reduction in a surgical setting in case of unexpected complications related to vital mediastinal structures that lie close to the SCJ. A closed reduction should only be performed on acute cases presenting within 48 hours of the initial injury.^[13]^ Attempting such a maneuver on a patient presenting more than 48 hours since the injury greatly reduces the success rates.^[9]^ After the reduction, the patient should be placed in a simple sling to allow for early mobility and avoid atrophy of muscular structures associated to the injured shoulder.^[20]^ However, closed reductions present a high potential for anterior instability resulting in high rates of joint redislocation.^[21,22]^ In addition, there may be fracture nonunion, due to the “blind” nature of the intervention, and exacerbation of an undetected intrathoracic injury.^[19]^ Given the low rates of success of closed reductions, some authors have suggested to directly attempt ORIF for the treatment of PSCJDs.^[19,23,24]^ There are multiple techniques available for the stabilization of the SCJ. These include costoclavicular cerclage with nonresorbable suture material,^[19]^ costoclavicular tenodesis using the subclavius muscle, sternoclavicular tenodesis using the sternocleidomastoid muscle,^[24]^ reconstruction of the SCJ using a semitendinous autograft placed in a figure-of-eight around the joint and placement of an LCP across the SCJ.^[12,25]^

Our case presents the added rarity and peculiarity of a physeal fracture in conjunction with a posterior displacement of the medial head of the clavicle. Given the promising results, we believe that ORIF represents a valid treatment option in the management of these types of injuries in adolescents.

## Author contributions

**Conceptualization:** Andreas Drossinos.

**Methodology:** Matteo Vitali.

**Project administration:** Matteo Vitali.

**Supervision:** Pierluigi Pironti, Vincenzo Salini.

**Validation:** Vincenzo Salini.

**Visualization:** Elisa Pesce.

**Writing – original draft:** Andreas Drossinos.

**Writing – review & editing:** Pierluigi Pironti.

Pierluigi Pironti orcid: 0000-0001-8458-7491.
